# Evaluation of ivermectin mass drug administration for malaria transmission control across different West African environments

**DOI:** 10.1186/1475-2875-13-417

**Published:** 2014-11-03

**Authors:** Haoues Alout, Benjamin J Krajacich, Jacob I Meyers, Nathan D Grubaugh, Doug E Brackney, Kevin C Kobylinski, Joseph W Diclaro, Fatorma K Bolay, Lawrence S Fakoli, Abdoulaye Diabaté, Roch K Dabiré, Roland W Bougma, Brian D Foy

**Affiliations:** Arthropod-borne and Infectious Diseases Laboratory, Department of Microbiology, Immunology and Pathology, Colorado State University, Fort Collins, CO USA; Department of Biomedical Sciences, Colorado State University, Fort Collins, CO USA; Walter Reed Army Institute of Research, 503 Robert Grant Ave, Silver Spring, MD USA; Armed Forces Research Institute of Medical Sciences, 315/6 Rajvithi Road, Bangkok, Thailand; US Naval Medical Research Unit No 3, Cairo, Egypt; Liberian Institute for Biomedical Research, Charlesville, Liberia; Institut de Recherche en Sciences de la Santé (IRSS), Direction Régionale de l’Ouest (DRO), Bobo Dioulasso, Burkina Faso; Ministry of Health, Ouagadougou, Burkina Faso

**Keywords:** Mass drug administration, Ivermectin, Transmission, West Africa, Malaria control, *Plasmodium falciparum*, *Anopheles gambiae*, Survival, Parity, Environment

## Abstract

**Background:**

Mass drug administration (MDA) of ivermectin to humans for control and elimination of filarial parasites can kill biting malaria vectors and lead to *Plasmodium* transmission reduction. This study examines the degree and duration of mosquitocidal effects resulting from single MDAs conducted in three different West African countries, and the subsequent reductions in parity and *Plasmodium* sporozoite rates.

**Methods:**

Indoor-resting, blood-fed and outdoor host-seeking *Anopheles* spp. were captured on days surrounding MDAs from 2008–2013 in Senegalese, Liberian and Burkinabé villages. Mortality was assessed on a portion of the indoor collection, and parity status was determined on host-seeking mosquitoes. The effect of MDA was then analysed against the time relative to the MDA, the distributed drugs and environmental variables.

**Results:**

*Anopheles gambiae* survivorship was reduced by 33.9% for one week following MDA and parity rates were significantly reduced for more than two weeks after the MDAs. Sporozoite rates were significantly reduced by >77% for two weeks following the MDAs in treatment villages despite occurring in the middle of intense transmission seasons. These observed effects were consistent across three different West African transmission dynamics.

**Conclusions:**

These data provide a comprehensive and crucial evidence base for the significant reduction in malaria transmission following single ivermectin MDAs across diverse field sites. Despite the limited duration of transmission reduction, these results support the hypothesis that repeated MDAs with optimal timing could help sustainably control malaria as well as filarial transmission.

**Electronic supplementary material:**

The online version of this article (doi:10.1186/1475-2875-13-417) contains supplementary material, which is available to authorized users.

## Background

Despite substantial efforts dedicated to control and eliminate malaria from certain regions, it is still a major public health issue. In 2012 nearly 207 million cases occurred with approximately 627,000 deaths, 77% of which were children younger than five years of age [1]. Current recommendations to combat malaria include artemisinin-based combination therapy (ACT) and long-lasting insecticidal nets (LLIN), supported by indoor-residual spraying of insecticide (IRS) and intermittent preventive treatment during pregnancy. Deployment of these strategies has fostered important reductions of malaria-associated morbidity and mortality in settings with moderate-to-high transmission levels in sub-Saharan Africa [2]. However, widespread insecticide resistance in vectors [3], increasing malaria cases in some African countries [1, 4], and concern over spreading artemisinin resistance [5], underlie the fragility of malaria prevention and control. To ensure the success of malaria elimination, the Roll Back Malaria Partnership and Malaria Eradication Research Agenda (malERA) consultative vector control group emphasized the need to search for innovative strategies [6]. These strategies should aim at developing new insecticides with novel modes of action, developing effective control methods for outdoor-feeding and resting vectors, and sustaining and integrating novel interventions in order to significantly decrease and even interrupt disease transmission in endemic areas.

**Figure 1:**
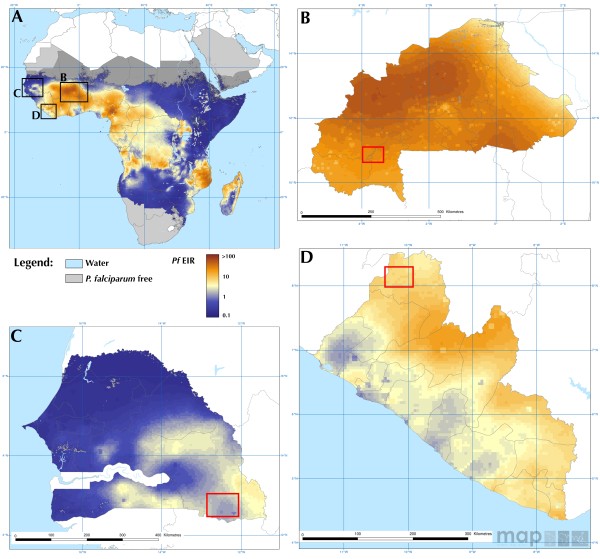
**Study sites outlined on maps showing the spatial distribution of the** ***Plasmodium falciparum*** **entomological inoculation rate (** ***Pf*** **EIR) in Africa (panel A), Burkina Faso (B), Senegal (C) and Liberia (D) in 2010.** Red squares represent the location of the sampled villages. Data are available at Malaria Atlas Project [13].

Ivermectin is an endectocide that has been extensively used alone for decades for the control of onchocerciasis, or in combination with albendazole for the elimination of lymphatic filariasis. Currently, more than 300 million individuals living in areas endemic for filarial infections are treated each year in mass drug administration (MDA) campaigns [7]. The drug has an excellent safety profile in humans and can be lethal to mosquitoes when they feed on treated humans. Ivermectin MDA addresses specific malERA recommendations including: a) a different mode of action from current insecticides; b) it targets all biting vectors, regardless of their ecology and feeding behaviour; and, c) it may be integrated into existing strategies to simultaneously control malaria, filariasis and other neglected tropical diseases [8]. The mosquitocidal effect of ivermectin MDA has been demonstrated on several *Anopheles* species from field trials [9, 10]. However, subsequent effects on vector population age-structure has only been modelled [11], and effects on vector infection rates with *Plasmodium* have only been measured for a limited duration in one setting [12]. Repeated MDAs with ivermectin have been proposed as a complementary *Plasmodium* transmission control tool [10] but several knowledge gaps need to be filled in order to fully evaluate this strategy. Here, the effects of single ivermectin MDAs were comprehensively analysed across different years and in three West African countries with distinct malaria transmission dynamics: Senegal, Liberia and Burkina Faso (Figure 1, Table 1). The degree and duration of effects on mosquito survival, parity rate and the proportion of sporozoite-infected vectors before and after single ivermectin MDAs in treatment villages, and in pair-matched, untreated villages, were assessed, taking into account several biotic (species, exophily) and abiotic (environmental) factors.

**Table 1:** **Characteristics of study sites**

Country	Phytogeographic zone	Rainy season	Malaria endemicity	MDA treatment	Diseases targeted
Burkina Faso	Sudanian	June-October	Hyperendemic	IVM + ALB	LF
Liberia	Tropical rainforest	Year round	Holo-endemic	IVM + ALB	LF, NTD, onchocerciasis,
Senegal	Sudano-Guinean	May-October	Hyperendemic	IVM	Onchocerciasis

## Methods

### Ethical statement

The study has been reviewed and approved by human subjects’ research reviews in each country (Senegal, *Etude des vecteurs du paludisme en zone onchocerquienne au Senegal*; Liberia, EC/LIBR/012/033; Burkina Faso, 28-2013/CE-CM) in compliance with the Helsinki Declaration. Informed consent was obtained from all adult and paid mosquito collectors. Human subject research protocols 11-3121H and 11-2874H were also approved by the Colorado State University Institutional Review Board.

### Study sites

Mosquito sampling was conducted during five collection periods in three West African countries: Senegal, Liberia and Burkina Faso (Table 1). *Senegal*: sampling occurred in 2008, 2009 and 2012 in the southeastern villages of Boundacoundi, Damboucoye, Nathia, Ibel, and Ndebou. Bed net coverage ranged from 78.2% in 2009 to 82.0% in 2012. Select villages in this region are treated by MDA with 150 μg/kg of ivermectin alone (Mectizan®, Merck & Co Inc) for onchocerciasis control. *Liberia*: sampling occurred in 2013 in the village of Ngaisaikoryah (Foya District, Lofa County) where the bed net coverage was 38.3%. MDA was designed to control lymphatic filariasis (LF), onchocerciasis and soil-transmitted helminths with a combination of ivermectin (150 μg/kg) + albendazole (400 mg) (Albenza®, GlaxoSmithKine). *Burkina Faso*: sampling occurred in 2013 in the villages of Bougouriba (Haut-Bassins region, control) and Diarkadougou East (Sud-Ouest region, treated) where the average bed net coverage was 42.0%. MDA was designed to control LF with a combination of ivermectin (150 μg/kg) + albendazole (400 mg). MDA coverage rates in treatment villages were 82.1-84.0% in Senegal, 76.2% in Liberia and 83.0% in Burkina Faso, and were recorded either directly by investigators or provided by health authorities.

### Mosquito collections and processing

Mosquito collections were performed by aspiration of indoor-resting, blood-fed mosquitoes on select mornings, as described by Sylla *et al*. [10]. Collected blood-fed females were kept in field insectaries, which were designated rooms of houses that had screened and slatted windows so that they naturally fluctuated with the ambient temperature and humidity. Temperature and relative humidity were monitored daily every 15 minutes in insectaries using a HOBO® device (Onset Computer Corporation) to determine the daily fluctuation of temperature and hygrometry, respectively. A subset of approximately 50 fully engorged *Anopheles gambiae s.l.* was transferred into cardboard cups covered with a mesh with access *ad libitum* to a sugar solution for survival analysis. Dead mosquitoes were counted daily, removed from cups over five consecutive days and identified morphologically to species. The head + thoracies of mosquitoes that survived after five days were stored individually in 1.5-ml tubes containing desiccant. Remaining collected mosquitoes were dissected on the same day of capture and head + thoracies were stored individually in 1.5-ml tubes with desiccant.

Additionally, outdoor host-seeking mosquitoes were captured over the night prior to morning house aspirations by either human-landing catch or tent traps, as previously described in Krajacich *et al.* [14]. These were identified morphologically to species and dissected for head + thoracies on the same day as capture as described above. A subset of these mosquitoes was also used for parity analysis.

### Parity determination

Age grading of *An. gambiae s.l.* was determined by parity analysis in samples from Senegal 2012 and from Burkina Faso 2013. A random batch of approximately 20 females from each village and each sampling day was dissected for their ovaries. Ovaries were dissected in water under a light microscope and allowed to dry. Parity rate was determined by observing the presence of coiled or uncoiled ovarian tracheoles [15].

### Mosquito species determination and *Plasmodium*spp. detection

DNA was extracted with the Qiagen DNeasy kit and was used to identify the species in the *An. gambiae* complex [16]. DNA from individual head + thoraxes were tested by Taqman polymerase chain reaction for *Plasmodium* spp. sporozoite detection [17], which used laboratory-confirmed *Plasmodium falciparum* sporozoite-infected *An. gambiae s.s.,* as positive controls.

### Statistical analysis

#### Mosquito survivorship

*Anopheles gambiae* survivorship was analysed using a generalized linear mixed-effect model with a binomial error structure to compare the effect of MDA between West African countries. The effect of eight variables on mosquito survivorship was analysed. These included the categorical variables field *sites* (Senegal 2008, 2009-mid season and 2009-end season, Liberia 2013 and Burkina Faso 2013), the *village* (treated or control), the *treatment type* (*ivm* or *ivm + alb*), the *species*, the *collection* (indoor or outdoor), the numerical variables *temperature* and *hygrometry fluctuations* (i.e., the difference between the maximum and the minimum values on each sampling day), and *time* relative to MDA date. The binary response variable was mosquito *survivorship*, counted as either dead or alive for each individual on the third day post-capture, when the maximal reduction in mosquito survivorship is observed [18]. Two types of analyses were performed: one to compare the difference in survival rate over time (in weeks) between treated and control villages and another to characterize the difference in survival rate over time (in weeks) between the treatment types. The first analysis was performed on all data obtained from Senegal and Burkina Faso only because there were no untreated villages during the sampling period in Liberia. This analysis included data from 3,140 *An. gambiae s. l.* and tested the influence of the site, village, species, collection, temperature and hygrometry fluctuations and time. The second analysis included data from 1,370 *An. gambiae s.l*., collected from treated villages only (including Ngaisakoryah, Liberia) and collected after the date of drug distribution only, and tested the influence of site, treatment type, species, collection, temperature and hygrometry fluctuation and time. The site variable was used as a random variable to account for the nested data structure, i.e*.*, the correlation between individuals from the same field site. For each analysis, the random structure was selected and compared with a generalized linear model with no random effect based on the lowest Akaike’s information criterion (AIC). Statistical analyses were performed with Statistical Analysis Software (SAS Institute Inc., Cary, NC, USA) using the GLIMMIX procedure. This procedure performs a type III hypothesis for the fixed effect variables and computes the *F*-statistic based on Satterthwaite’s approximation. The maximal models included all the variables with interactions and were subsequently simplified following a step-by-step AIC-based procedure. Output of the minimal model was used to produce the local regression (LOESS procedure) with the computed 95% confidence intervals. The mean survival rates by week were computed and compared between villages (control and treated) taking into account multiple testing using the Bonferroni procedure.

#### Parity rate

Parity data were analysed weekly and significant differences in parity rate over time were assessed separately for the control (N = 327) and the treated (N = 380) villages, and also between villages for each week, separately (pre-MDA: N = 222, week 1: N = 130, week 2: N = 112, week 3: N = 243) using the χ^2^-test. The parity data were analysed also with a generalized linear model with a binomial error structure (GLM procedure in SAS) to produce the local regression (LOESS procedure) with the computed 95% confidence intervals.

#### Sporozoite rate

Variations in sporozoite rates over time were analysed only from data obtained in Senegal 2012, Liberia 2013 and Burkina Faso 2013 reported here. Analysis was performed using a generalized linear model with a binomial error structure (GLM procedure in SAS), due to the low number of field sites included. The effect of seven variables on mosquito sporozoite rate was analysed: site, village, species, collection as categorical variables and temperature and hygrometry fluctuations and time relative to MDA (in weeks) as numerical variables. The binary response variable was the status of *Plasmodium* infection in thoraces (i.e., infected or uninfected). The model was subsequently simplified following a stepwise AIC-based procedure. Significance of the variable retained in the minimal model was determined using the type III test and normality of residuals was checked. Output of the minimal model was used to produce the local regression (LOESS procedure) with the computed 95% confidence intervals.

## Results

### Effect of MDA on *Anopheles gambiae*survival rate

The three-day survival rate of *An. gambiae s.l.* collected in each field site is summarized in Table 2 by sampling week. The three-day survival rate was first analysed in treated villages and compared with that from controls, so only data from Burkina Faso and Senegal (2008 and 2009) were used because a control village in Liberia was not sampled. The influence of field sites appeared to be weak as the variance of the random variable site is 0.05571 ± 0.05186 (estimate ± standard error), but still distinct from zero. Mosquito survival rate was significantly influenced by the variables: village (*F*_1,3434_ = 5.48, *p* = 0.0193), time (*F*_2,3434_ = 21.22, *p* < 0.001), hygrometry fluctuation (*F*_1,3434_ = 9.66, *p* = 0.0019; Additional file 1: Figure S1), and the interaction of village with time (*F*_2,3434_ = 13.62, *p* < 0.001). Neither the species nor the exophily were retained in the minimal model. The computed three-day survival rate of *An. gambiae s.l.* over time in the treated and the control villages from all field sites shows a reduction during the first week after MDA from 82.3% ±2.0 in the control villages to 54.4% ±3.2 in the treated villages (a 33.9% reduction over the three days). Hygrometry fluctuation negatively influenced the mosquito survival rate (Spearman correlation coefficient; -0.5253, *p* < 0.0001; Additional file 1: Figure S1).

**Table 2:** **Three-day survival rate of wild caught** ***Anopheles gambiae s.l.*** **following mass drug administration of ivermectin in the treated village compared to the control village**

Study site	Year	Village	Time relative to MDA (week)					
			Week -3	Week -2	Week -1	Week 1	Week 2	Week 3
Burkina Faso	2013 (Aug.-Sept.)	Control	0.96 ± 0.02 (89)	0.70 ± 0.05 (70)	NA	0.71 ± 0.03 (266)	0.651 ± 0.04 (129)	0.89 ± 0.05 (45)
		Treated	0.85 ± 0.04 (75)	0.75 ± 0.04 (122)	NA	0.50 ± 0.03 (329)	0.709 ± 0.03 (196)	0.81 ± 0.06 (41)
		*p-value*	***0.0477***	*0.6029*	-	***1.54E-07***	*0.3268*	*0.4325*
Liberia	2013 (June)	Control	NA	NA	NA	NA	NA	NA
		Treated	NA	0.98 ± 0.01 (104)	0.91 ± 0.04 (66)	0.42 ± 0.04 (147)	0.86 ± 0.05 (50)	NA
		*p-value*	-	-	-	-	-	-
Senegal	2009 (Oct.)	Control	NA	NA	0.83 ± 0.06 (40)	0.81 ± 0.05 (62)	0.792 ± 0.08 (24)	NA
		Treated	NA	NA	0.79 ± 0.02 (338)	0.48 ± 0.04 (168)	0.75 ± 0.06 (48)	NA
		*p-value*	*-*	*-*	*0.7879*	***1.5E-05***	*0.9218*	*-*
	2009 (July-Aug.)	Control	NA	0.77 ± 0.08 (26)	0.61 ± 0.09 (33)	0.72 ± 0.06 (60)	0.833 ± 0.07 (30)	NA
		Treated	NA	0.90 ± 0.04 (59)	0.82 ± 0.03 (154)	0.57 ± 0.04 (150)	0.819 ± 0.03 (160)	NA
		*p-value*	-	*0.2162*	***0.015***	***0.077***	*1*	-
	2008 (Aug.)	Control	NA	NA	0.76 ± 0.07 (37)	1.0 ± 0 (26)	0.842 ± 0.08 (19)	NA
		Treated	NA	NA	0.70 ± 0.05 (89)	0.40 ± 0.06 (70)	0.636 ± 0.15 (11)	NA
		*p-value*	*-*	*-*	*0.6427*	***4.78E-07***	*0.4031*	*-*

Data presented are mean survival rate ± s.e. with sample size in brackets. Significance of differences (*p*-value) between the control and the treated villages were assessed using χ^2^ test, *p*-value below 0.05 are indicated in bold.

### Comparison of MDA treatments on the survival rate of *Anopheles gambiae*

This analysis focused only on treated villages to characterize the duration of the reduction in survival rate post-treatment, as well as the potential effect of the different drug regimens (either ivermectin + albendazole or ivermectin alone), thus it included data from all three ecological settings starting from the date of MDA. As expected, the influence of time is significant because survival rate increased over time to the pretreatment level (*F*_1,1361_ = 73.72, *p* < 0.001). Interestingly, the effect of time was different between treatment types (*treat. type* by *time* interaction: *F*_1,1361_ = 5.36, *p* = 0.0208), suggesting that the ivermectin + albendazole treatment may have resulted in an apparent longer-lasting mosquitocidal effects as compared to treatment with ivermectin alone. However, the degree of reduction in mosquito survival rate did not differ between treatment types: from an average of 88.2% ±2.6 before MDA to 53.44% ±3.4 for ivermectin alone, compared to 59.69% ±2.4 for ivermectin + albendazole, the week following MDA (*p* = 0.809). The hygrometry fluctuation did not significantly influence the overall survival rate (*F*_1,1361_ = 0.22, *p* = 0.6427), but the analysis revealed a significant interaction of hygrometry variation with treatment type (*Hygro.* by *Treat.* interaction: *F*_1,1361_ = 5.85, *p* = 0.0157).

### Parity rate

**Figure 2:**
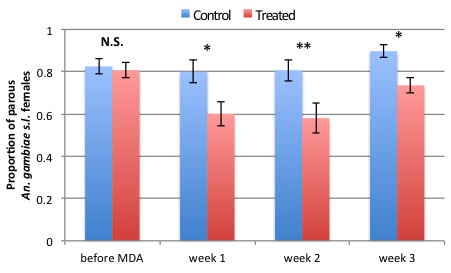
**Mosquito parity rate over time in treated and control villages.** Significant differences in parity rates between the control (blue bars) and the treated villages (red bars) are denoted by stars above the column pairs and derived using a Chi-squared test (N.S. = not significant). Error bars are the standard error of the mean. Sample sizes were 103 and 119 pre-MDA, 55 and 75 on week 1, 62 and 50 on week 2 and 107 and 136 on week 3 in the control and the treated villages, respectively.

Figure 2 represents the variation in parity rate by week and shows no significant variation over time in the control village (χ^2^_df=3_ = 3.96, *p* = 0.265, N = 327), ranging between 80 and 89.72% (mean of 85.4% ±2.3). In contrast, the proportion of parous female *An. gambiae s.l.* varied significantly over time in the treated village (χ^2^_df=3_ = 14.36, *p* = 0.0024, N = 380). While 80.7% ±3.6 of host-seeking females were parous before MDA in the treatment village and showed no significant difference with the control village (χ^2^_df=1_ = 0.033, *p* = 0.856, N = 222), this proportion significantly decreased to 60.0% ±5.6 (χ^2^_df=1_ = 4.98, *p* = 0.0255, N = 130) over the first week post-MDA and to 58.0% ±7.0 (N = 50, χ^2^_df=1_ = 5.78, *p* = 0.0161, N = 112) over the second week post-MDA. By the third week post-MDA, the parity rate in the treated villages increased to 73.5% ±3.8 but remained significantly lower than in the control villages (χ^2^_df=1_ = 9.05, *p* = 0.0026, N = 243).

### Sporozoite rate

A proportion of all vectors were processed on the day of capture from Senegal 2012, Liberia 2013 and Burkina Faso 2013 (Additional file 2: Table S1) and analysed for sporozoite infections over time. Overall, the variable time did not significantly influence the sporozoite rate (χ^2^_df=5_ = 8.198, *p* = 0.1456). However, the variation across time is significantly different between villages (*time:village* interaction: χ^2^_df=5_ = 25.89, *p* < 0.001), indicating a difference between control and treated villages. The mean sporozoite rate in the control villages did not significantly change over time, varying from 2.71% ±0.8 to 4.84% ±0.9 (*p* = 1). In the treated villages, ivermectin MDA reduced the sporozoite rate from 5.31% ±1.2 pre-MDA to 2.03% ±0.8 the first week (*p* = 0.0074) and to 1.19% ±0.7 the second week after MDA (*p* = 0.0018). In addition, the significant *site*:*time* interaction (χ^2^_df=5_ = 29.25, *p* < 0.001) indicated that the variation of sporozoite rate across time is significantly different between malaria ecologies. However, the minimal model did not retain the *time*:*site*:*village* triple interaction, indicating that the difference between control and treated villages over time is consistent among field sites. Interestingly, sporozoite rate was also significantly influenced by daily temperature fluctuation (χ^2^_df=1_ = 7.334, *p* = 0.0068; Additional file 3: Figure S2).

### Combined mosquito survival, parity and sporozoite rate over time

**Figure 3:**
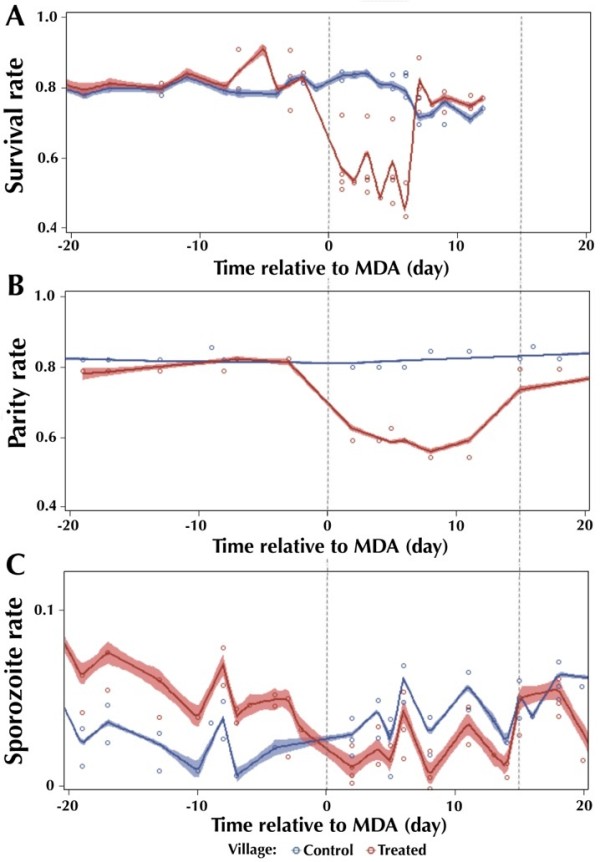
**Dynamic of survival (A), parity (B), and sporozoite (C) rates across time relative to mass drug administration.** Daily data points represented as circles from control (blue) and treated (red) villages, with the computed 95% confidence intervals using the local regression method (LOESS) in SAS software.

Figure 3 shows the dynamic of survival, parity and sporozoite rates across time (individual sampling days) relative to the MDA date, obtained by local regression of all relevant data. For most of the pre-MDA sampling dates, the control and treated villages do not significantly differ in survival rate (Figure 3A) and parity rate (Figure 3B) as evidenced by overlapping 95% confidence intervals. Over this same time interval, the mosquito sporozoite rate (Figure 3C) from treated villages was significantly greater than from pair-matched control villages, and fluctuated more significantly between sites and villages, as evidenced by more broad 95% confidence intervals, likely due to the variable nature of human-to-mosquito transmission. Immediately following MDA in the treated villages, all three measures were significantly reduced in the treated villages compared to control villages. Survival rate in treated villages was the first measure to recover to pre-MDA and control village values, at day 7 post-MDA. The parity rate never recovered to pre-MDA and control village values by the end of the sampling, but it did hit a slower increasing plateau after 15 days post-MDA (dotted line on the right of all graphs). On this same date post-MDA, the sporozoite rate in treated villages recovered to the same rate as pre-MDA and control villages.

## Discussion

In an attempt to comprehensively evaluate the effects of single ivermectin MDA, alone or in combination with albendazole, on malaria transmission, their effect on natural populations of mosquito vectors was characterized in three West African countries with different malaria ecologies. The primary first order effect of ivermectin MDA was on mosquito survival rate, which was consistent across all sites. While lasting a relatively short time (one week), the observed lethal effect of ivermectin on the vector population biting the villagers is strong with respect to vectorial capacity. The calculated reduction in daily mosquito survival rate (*p*) of 11% would lead to a 78% reduction in vectorial capacity for the week following the MDA [19]. Nonetheless, recent modelling of the impact of ivermectin on malaria transmission demonstrated that the duration of the mosquitocidal effect has a greater impact than its magnitude [20]. The two different drug regimens did not differently affect the degree of mosquito survival rate change, suggesting no additional effect from albendazole, which is consistent with published laboratory results [18]. While differences in field sites did not appear to strongly influence the model, this analysis seems to indicate that the ivermectin + albendazole regimen resulted in a longer mosquitocidal effect compared to the ivermectin alone regimen (significant *treat. type* by *time* interaction). Awadzi *et al.* [21] found no significant differences in ivermectin plasma pharmacokinetics when administered with albendazole, potentially ruling out drug-drug interactions in humans, however drug-drug interactions in the mosquito might be a potential factor. From this study, it is not clear what is influencing this observation and further experiments are needed to characterize the role of other biotic or abiotic factors affecting vector population susceptibility.

Modelling predicted that the mortality effects from a single MDA would temporarily shift the population structure of vector mosquitoes around treated villages [11]. In concordance with this model, the proportion of parous, outdoor host-seeking mosquitoes in the populations studied was reduced by 25%. This reduction is equivalent to parity rate changes observed in *An. gambiae* populations after implementation of IRS with dichlorodiphenyltrichloroethane (DDT) [22] and LLIN distribution [4]. The observed reduction in parity rate may, in part, be due to an increased susceptibility to ivermectin in older mosquitoes, a phenomenon that has been reported with the use of other insecticides, including pyrethroids and DDT [23]. Such an effect would lead to a greater impact on malaria transmission, as mosquito vectors need to survive the extrinsic incubation period to become infectious (approximately ten days). The one-week reduction of survival rates resulted in a significant shift to a younger mosquito population that lasted more than three weeks after the MDA. This suggests: a) that ivermectin concentrations found in human blood after the first week could still selectively kill older and/or infected mosquitoes, and/or, b) that the population returns to its initial age-structure at a relatively slow speed. In line with the first hypothesis, Kobylinski *et al.* showed that sublethal concentrations of ivermectin reduced the proportion of *P. falciparum* (NF-54 strain)-infected *An. gambiae* at both oocyst and sporozoite stages, suggesting an increased susceptibility of infected mosquitoes [24].

Consistent across field sites, sporozoite rates were reduced by 77.5% for 15 days. The observed sporozoite rate reductions is likely explained by the combination of various ivermectin effects against factors influencing vectorial capacity, including: a) mosquito survival rate (with possible selective effects against older and/or *P. falciparum* infected adults); b) vector density relative to hosts (effects on egg-laying ability [25]); c) time between mosquito blood meals (refeeding frequency [18]); and, d) potential effect on vector competence (i.e., ability of vectors to support parasite development). The change in sporozoite rate over time reflects the change in parity rate over time, suggesting that the shift of the age-structure may be the main cause of the reduction of sporozoite prevalence in mosquito vectors. While laboratory studies have suggested an anti-sporogonic effect (inhibition of parasite development in the vector) [24], ongoing semi-field and field studies examining the effect of ivermectin on wild-type mosquitoes and parasites will need to sort out these discrepancies.

The limitations of this study include some unavoidable inconsistencies in sampling and processing regimens across each year and field site; for example, a control village was not concomitantly sampled in Liberia. Even so, a single ivermectin MDA has a clear and dramatic impact on the proportion of infectious mosquitoes for up to 15 days. As previously proposed, repeated ivermectin MDAs would be necessary to have a sustained effect on *Plasmodium* transmission [8, 11]. Repeated ivermectin MDAs have been shown to be safe, even at higher doses than currently administered [26]. Such regimens also have the potential to integrate with control of neglected tropical diseases (NTD) such as onchocerciasis, LF, soil-transmitted helminths (strongyloidiasis, ascariasis, trichuriasis, and hookworm), scabies, and lice [27]. Questions remain about how frequent, repeated, ivermectin MDAs would need to occur with respect to when in the transmission season they would be administered. The maximal effect would probably occur if MDAs were concentrated during dry-to-wet season transitions, when the numbers of vectors are low but increasing, and thus most susceptible to population level effects. This may also have the most significant effect on malaria incidence, as these transition seasons are also concomitant with peak seasonal risk of clinical malaria in children living in hyperendemic areas, likely due to acquisition of new *Plasmodium* clones when mosquito biting increases [28]. While repeated ivermectin MDAs would reduce *Plasmodium* transmission, this effect alone would not reduce or eliminate the human reservoir of *Plasmodium* parasites. Integration with other control measures is critical. Modelling has shown that combining repeated ivermectin MDAs with anti-malarial drugs (e.g., ACT) would sustain transmission interruption and achieve malaria elimination [20]. Addition of albendazole with ivermectin during MDAs would maximize impact against NTDs and help prevent development of helminth resistance to either drug [29]. Lastly, community-directed treatment platforms for ivermectin delivery have been shown to more cost-effectively and efficiently deliver LLINs [30, 31]. A large-scale randomized trial would be required to fully evaluate the implementation of multiple control methods alone or in combination. It would be expected that ivermectin MDAs would synergize with LLIN and IRS control methods to more significantly reduce the proportion of infectious vectors, especially in areas where pyrethroid/DDT resistance in vectors is common. Ivermectin MDAs might particularly help to control malaria transmission from outdoor-biting vectors that are less affected by the latter control methods. Overall, such integrated strategies would control many diseases simultaneously, and should lead to strong and lasting health benefits in the treated communities.

## Conclusions

These data show that single ivermectin MDAs significantly affect mosquito population survival rates, which temporarily changes mosquito population age-structure and results in significantly suppressed sporozoite rates for 15 days after the MDA. The results were collected across multiple years and diverse West African malaria ecologies, and demonstrate a consistent effect despite these differences. These data provide a strong evidence base to develop repeated ivermectin MDA for malaria control and elimination strategies, especially when integrated with complementary malaria and NTD control strategies that all can utilize the very successful community-directed treatment models developed for onchocerciasis and LF control.

## Electronic supplementary material

Additional file 1: Figure S1: Correlation between *Anopheles gambiae s.l.* survival rate and hygrometry fluctuation (dH). Shaded area around the regression line is 95% CI. Spearman correlation coefficient: -0.5253, *p* < 0.001. Dots indicate data from distinct field sites: light green: Senegal 2008, pink: Senegal July-Aug. 2009, dark green: Senegal Oct. 2009, red: Burkina Faso 2013. (JPEG 3 MB)

Additional file 2: Table S1: Sporozoite-infected malaria vectors collected indoor and outdoor. Sample sizes are indicated in brackets. HLC, LTC refer to human-landing catch and light trap catch, respectively. (XLSX 45 KB)

Additional file 3: Figure S2: Correlation between *Anopheles gambiae s.l.* sporozoite rate and temperature fluctuation (dT). Shaded area around the regression line is 95% CI. Pearson correlation coefficient: 0.38360, *p* < 0.001. Dots indicate data from distinct field sites: green: Senegal 2012, blue: Liberia 2013, red: Burkina Faso 2013. (JPEG 3 MB)

## Abbreviations

MDA: Mass drug administration
ACT: Artemisinin-based combination therapy
LLIN: Long-lasting insecticidal nets
IRS: Indoor-residual spraying
malERA: Malaria eradication research agenda
LF: Lymphatic filariasis
IVM: Ivermectin
ALB: Albendazole
AIC: Akaike’s information criterion
GLM: General linearized model
DDT: Dichlorodiphenyltrichloroethane
NTD: Neglected tropical diseases
*Pf*EIR: *Plasmodium falciparum* entomological inoculation rate.
