# Supplementary figures and images for: Evaluation of ivermectin mass drug administration for malaria transmission control across different West African environments
Source: Malar J. 2014 Nov 3;13:417. doi: 10.1186/1475-2875-13-417 (PMC4226880; doi:10.1186/1475-2875-13-417)

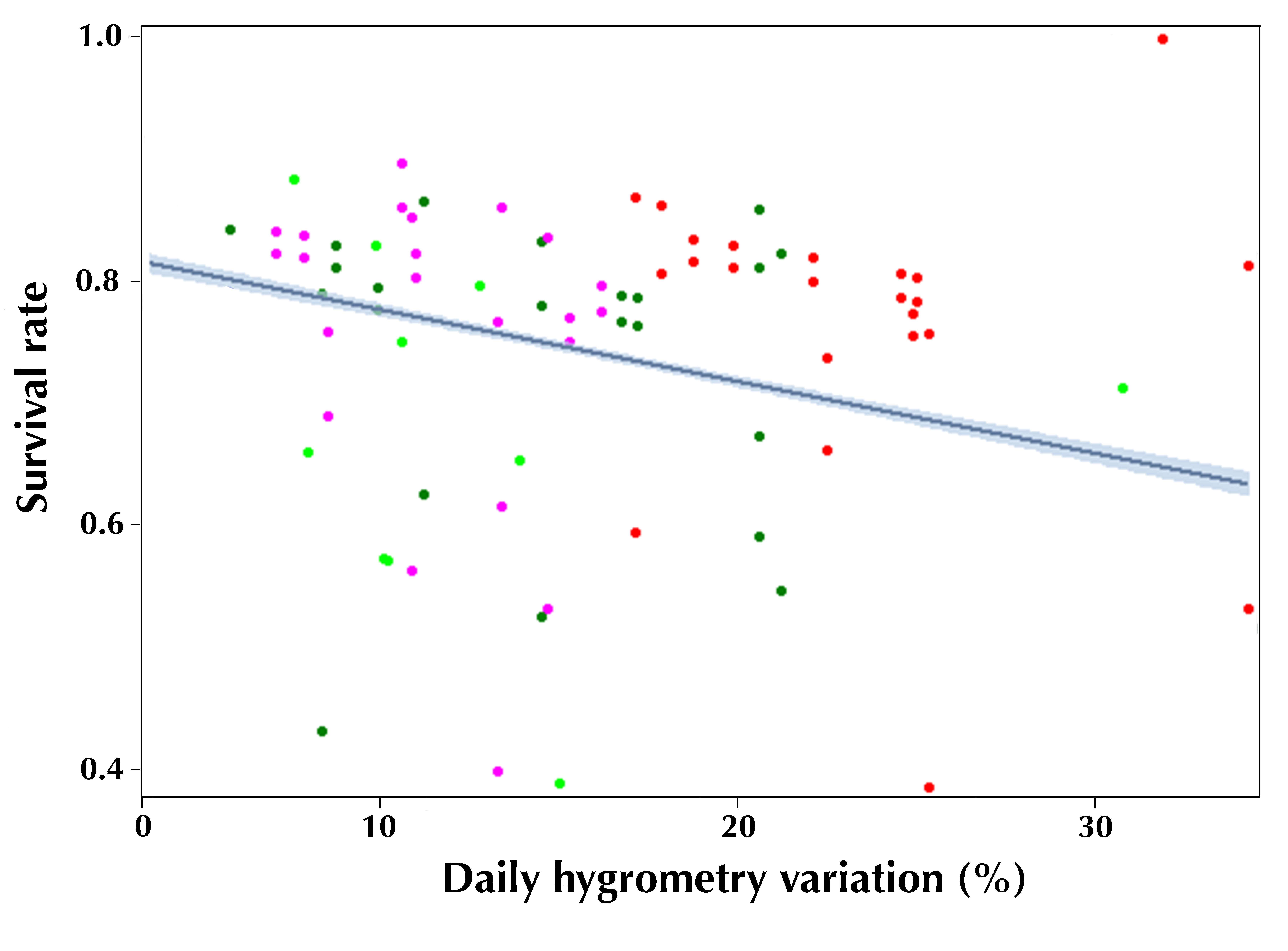

Supplement: Supplementary file 1 — Additional file 1: Figure S1: Correlation between Anopheles gambiae s.l. survival rate and hygrometry fluctuation (dH). Shaded area around the regression line is 95% CI. Spearman correlation coefficient: -0.5253, p < 0.001. Dots indicate data from distinct field sites: light green: Senegal 2008, pink: Senegal July-Aug. 2009, dark green: Senegal Oct. 2009, red: Burkina Faso 2013. (JPEG 3 MB) [file 12936_2014_3580_MOESM1_ESM.jpeg]

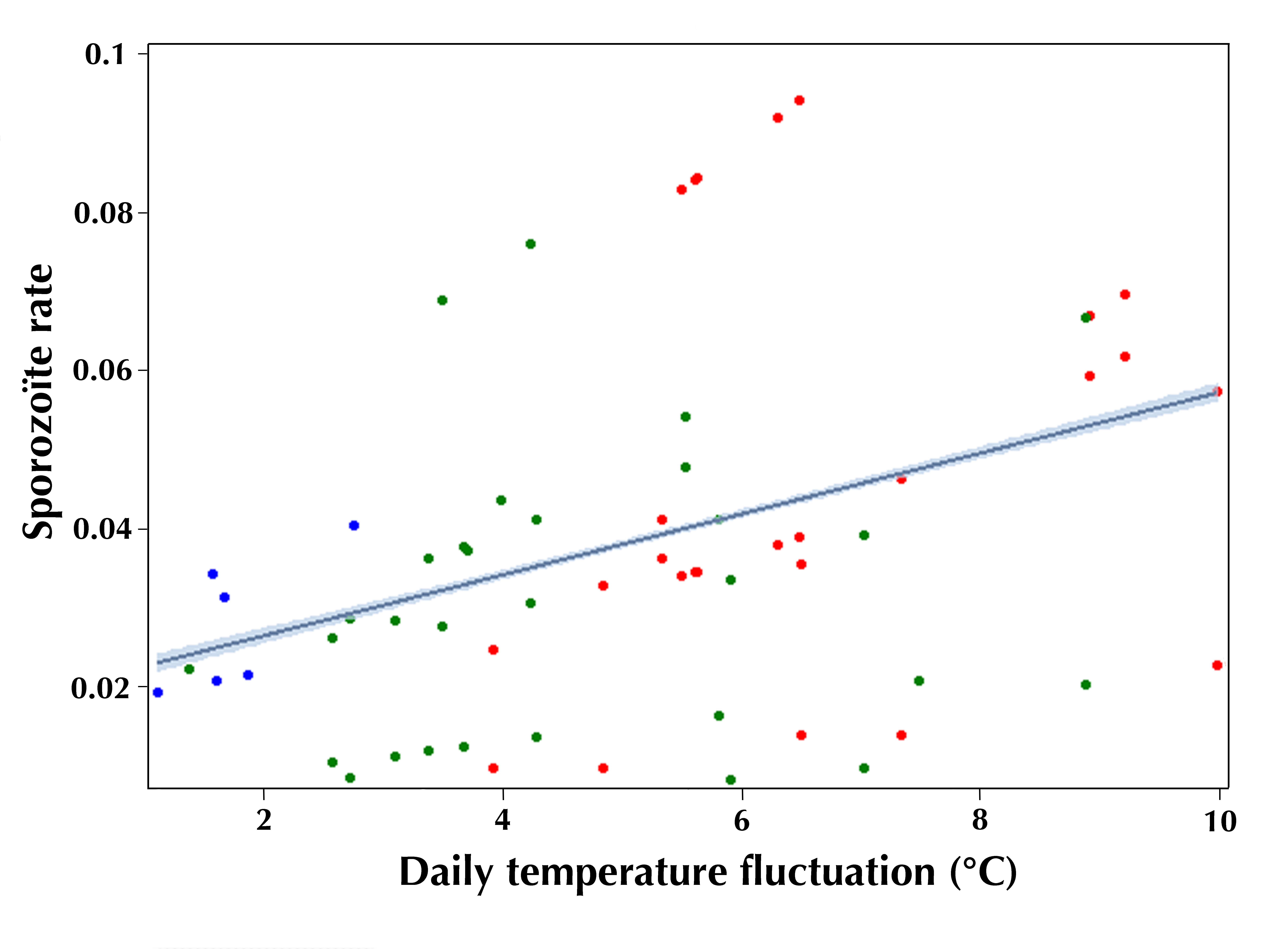

Supplement: Supplementary file 3 — Additional file 3: Figure S2: Correlation between Anopheles gambiae s.l. sporozoite rate and temperature fluctuation (dT). Shaded area around the regression line is 95% CI. Pearson correlation coefficient: 0.38360, p < 0.001. Dots indicate data from distinct field sites: green: Senegal 2012, blue: Liberia 2013, red: Burkina Faso 2013. (JPEG 3 MB) [file 12936_2014_3580_MOESM3_ESM.jpeg]
